# How Does Public Service Motivation Affect the Proactive Service Behaviors of Grid Workers? A Study of Survey Evidence from Eastern China

**DOI:** 10.3390/bs14030148

**Published:** 2024-02-20

**Authors:** Lijun Chen, Chuanxue Lin, Xiaorui Zhou

**Affiliations:** 1 School of Public Affairs, Zhejiang University, Hangzhou 310058, China; lijunchen@zju.edu.cn; 2 Zhejiang Institute of Talent Development, Hangzhou 310028, China

**Keywords:** Chinese grid workers, proactive service behaviors, public service motivation, occupation identity, organizational support, organizational climate

## Abstract

In China, grid workers have increasingly become an indispensable and important force in basic social governance. They not only undertake several tasks, such as gaining publicity, collecting information, resolving conflicts, and assisting in management, but they also actively serve the grid residents enthusiastically and engage in proactive service behaviors. In order to better cultivate this important force, we hope to have a better understanding of the factors contributing to the behavioral performance of grid workers, especially the impact of organizational and personal factors. In this study, we sought to establish what factors influence the proactive service behaviors of grid workers. Based on a theoretical consideration of factors such as public service motivation, occupational identity, and organizational climate, a multi-factor influence hypothesis model was constructed to explain the proactive service behaviors of these workers. By analyzing data based on 348 paired survey samples received in two stages in eastern China, these hypotheses were then tested. The results reflect that grid workers’ public service motivation can stimulate proactive service behaviors. Furthermore, occupational identity plays a mediating role, while organizational support and organizational service climate play a positive moderating role between public service motivation and occupational identity. This finding clarifies the important influencing factors of proactive service behaviors among grassroots workers, such as grid workers, and has important implications for how to effectively motivate these groups to provide more proactive services, promoting their sustainable development and improve the effectiveness of grassroots governance.

## 1. Introduction

Since grid governance was first introduced in Beijing, China in 2004, this governance model has been widely implemented in other Chinese cities [1], and it plays a huge role in basic social governance in China. Grid governance refers to the division of urban communities and villages into several grids based on their geographic and administrative boundaries, and the embedding of affairs, organizations, and people into specific grids [1,2]. Within each grid, one or more grid workers are assigned, and the head of a grid—the person who coordinates matters within the grid and is responsible for communicating with the community, street office, etc.—is called the “grid leader”. Therefore, the vast number of grid workers is the core vehicle of grid governance in China.

Under the grid-governance model, the grid workers in each grid are responsible for several tasks, such as information collection, investigation of hidden dangers, and conflict mediation [2]. During the COVID-19 epidemic, China fully mobilized its grid workers and adopted various grid-based governance measures. This enabled residents’ lives and factories’ production to gradually return to normal, effectively demonstrating the important role of grid-based governance in containing the epidemic [3]. However, the occupational group of grid workers appeared relatively late in this process. Furthermore, most of the personnel involved in this group were people outside the authorized personnel quota in the public sector, which is different from the group of Chinese civil servants in terms of treatment and social status. At the same time, grid workers are mainly active at the grassroots level, and this complex working environment means that they will face a series of uncertainties and dilemmas. In terms of work requirements, the two main assessment indicators for grid workers are: result-oriented indicators, which consider the governance of their grids; and behavioral indicators, which are more process-oriented but are only minimally reflected in assessments.

The behavior of Chinese grid workers under poor work remuneration has caused us confusion. According to JD-R theory, adequate resources can stimulate employees’ motivation, increase their work engagement, and thus bring positive organizational results [4]. Correspondingly, high job demands and insufficient work resources will lead to job burnout, as well as negative impacts on organizations and individuals. However, through empirical observation, we have found that many grid workers not only complete corresponding work tasks, but also provide many warm and proactive services. In many news reports, touching examples of China’s grassroots workers continue to emerge, and, even during the worst times of an epidemic, grid workers have always stepped up to the plate. In the concrete practice of Chinese grid workers, some are proactive, dedicated, and enthusiastic in their service and can spontaneously contribute to the optimization and improvement of the grassroots governance of their grids beyond the requirements of their work. They not only fulfill the basic assessment requirements but also take the initiative to provide humane care throughout their work. For example, they continue to monitor the living conditions of the elderly in their grids after work, take the initiative to give up their rest time to stay on the front line, and pay attention to their words and actions during the service process; this results in significant convenience for and thoughtful service to people at the grassroots. Stories of grid workers’ proactive service appear frequently in news reports, suggesting that proactive service behaviors are indeed widely manifested in the group of grid workers.

Why can grid workers still exhibit these proactive service behaviors in busy work situations? We cannot help but think about the causes behind these proactive service behaviors, especially considering the impact of personal and organizational factors, as this may affect the actual work conditions of grid workers and determine whether this proactive service behavior can be sustained. In the context of Chinese culture, we are well aware that directly discussing the work status of grid workers, including considering their work happiness, may not be effective. Due to factors such as social desirability, these grassroots workers may not necessarily express their true thoughts, but their work status may be perceived from their behaviors. In related research on organizational behavior, empirical work has found a correlation between individual affective commitment and proactive service behaviors [5,6]. Studies have shown that employees’ emotions have a significant impact on their proactive behaviors, and proactive behaviors may come more from emotions than from rationality. For example, Fritz and Sonnentag found that positive emotions can trigger proactive behaviors in individuals [7]. In the active motivation model, the “passion” pathway, which reflects positive emotional states, is also a key factor in generating proactive behaviors [8]. In this pathway, individual intentions, when transformed into positive psychological states, are effective in generating positive behaviors. Therefore, we attempt to understand the work status of this professional group from the proactive service behavior of grid workers. Another background factor that supports us continuing research is that we follow the United Nations Sustainable Development Goals initiative; we especially focus on “health and wellbeing at work” (SDG-3) and “decent work and economic growth” (SDG-8) (Available online: https://www.un.org/sustainabledevelopment/zh/sustainable-development-goals/ (accessed on 8 February 2024)). The United Nations Sustainable Development Goals is a collection of 17 global goals for transforming our world by 2030, published and adopted by the United Nations in 2015. The Sustainable Development Goals aim to comprehensively address development issues in the three dimensions of society, economy, and environment from 2015 to 2030, and create a shift towards a path of sustainable development. Through our research, we hope to better understand this working group and help them achieve sustainable development.

In academic research, the concept of proactive service behavior is defined as individuals’ self-started, long-term-oriented, and persistent service behavior that goes beyond explicitly prescribed performance requirements [9]; this is consistent with the community-service behaviors of grid workers. Studies of proactive service behavior were developed from research related to proactive behavior. Since the 1990s, a large number of scholars in the field of organizational behavior have explored the conceptualization of proactive behavior [10,11,12,13,14]. Proactive behavior is defined as “taking initiative in improving current circumstances or creating new ones, and it involves challenging the status quo rather than passively adapting to present conditions” [14,15]; employees engaging in proactive behavior can invest in their work with passion and put notable effort into achieving their desired outcomes [16].

Fay and Frese [13] discussed the general characteristics of proactive behaviors from the perspective of different kinds of personal initiative, including self-starting, pro-active, pro-company and persistent initiative. Rank’s definition of the three characteristics of proactive service behaviors is based on this theoretical foundation; his research team identified a lack of research on proactive behaviors in the service sector [9]. At the time, the potential positive impact of the behaviors of frontline service employees were attracting attention in the academic community [17,18]. Therefore, the concept of proactive behavior was creatively transplanted to the service field, and the concept of proactive service behaviors in frontline service workers was proposed based on the service orientation of individuals. Rank’s conceptual innovation of proactive service behavior not only effectively connects the research contents of different fields but also expands the corresponding theoretical perspectives [9]. Indeed, proactive service behaviors are at the intersection of two fields of research: one explores the specific manifestations of a large number of proactive behaviors in different practice contexts, ignoring their significance for the service domain, while the other focuses on how the performance of service providers improves the customer experience [19]; however, this fails to consider the dynamic role played by individual initiative.

Being at the grassroots level in China, grid workers are required to participate in all kinds of affairs within their grids on a daily basis, and they have a great deal of interaction with the people within their grid areas, so their occupational status has service attributes. Cities in China have also incorporated the qualities of accessibility and service to the community into their recruitment announcements for grid workers, suggesting that a qualified grid worker should have a certain level of service capability. Through proactive service behaviors, grid work can improve the level of community service from the perspective of residents. Furthermore, the self-started and long-term-oriented characteristics of proactive service behaviors coincide with the needs of grassroots grid governance, and they can also respond to the public’s increasing demands for better services from grid governance. Strengthening grid workers’ proactive service behaviors will help the governance of the grid in which they are located.

This study focused on the proactive service behaviors of Chinese grid workers and their possible influencing factors, and it sought to provide more research evidence for the study of proactive service behaviors. The findings of this study may also provide theoretical guidelines for improving the performance of the role of this particular governance team. Due to the changes in the work context in which the research participants are located, this study also sought relevant variables that fit the group’s work characteristics. According to Crant’s research [14], the antecedent variables of proactive service include both individual differences and contextual factors. Among these, individual differences include personality factors, such as individual motivation, while contextual factors mainly include factors related to the external environment, such as organizational culture.

Therefore, several individual characteristics and contextual factors were introduced as variables in this study, and a variable relationship model of grid workers’ proactive service behaviors was constructed based on several research hypotheses. These hypotheses were then tested using the results of paired questionnaires.

## 2. Theory and Research Hypotheses

### 2.1. Public Service Motivation and Proactive Service Behavior

To examine the personal motivational factors affecting the proactive service behaviors of grid workers, we considered the concept of public service motivation (PSM). PSM was first introduced by Perry and Wise in 1990 [20]; they defined PSM as “an individual’s predisposition to respond to motives grounded primarily or uniquely in public institutions and organizations.” Research on the concept of PSM has demonstrated that this individual-level motivation is largely altruistic and rooted in the public sector [21]. Based on a conceptual discussion, Perry classified the content of PSM into four dimensions: attraction to public policymaking, commitment to civic duty and the public interest, compassion, and self-sacrifice [22]. The connotations of PSM have since been extended, with an increasing number of studies demonstrating increasing attention from scholars and policy practitioners [21,23]. In China, where the work purpose of “serving the people” (为人民服务) has been deeply rooted in people’s minds for a long time, researchers have carried out a large number of studies based on the theory of PSM [24,25,26,27].

PSM has a strong connection to proactive service behavior; for example, both concepts are used to describe proactive behavior [9,20]. However, the difference lies in the fact that whereas PSM describes the motivation of public-sector personnel to make proactive sacrifices for the public good—especially sacrifices of pecuniary gain—proactive service behavior tends to describe the characteristics of this proactive behavior [22,28]. Indeed, Crant argues that an individual’s proactive personality is an important antecedent variable of proactive behavior [14]. Luu found that PSM plays a reinforcing role in the effect of discretionary human-resources practices on proactive work behavior, playing a reinforcing role; this confirms the existence of a positive effect of PSM on individual proactive behavior [5].

The PSM of grid workers is focused on public-interest commitment, empathy, and selflessness; this is centered on caring for the public, and it thus stimulates proactive service behaviors toward grid residents, corresponding to the “willingness” pathway in the proactive motivation model [29]. Based on the “motivation–behavior” pathway in organizational behavior research, we hypothesize that the proactive service behavior of grid workers may be influenced by their intrinsic motivation to serve, which in turn leads to proactive service behaviors. The following hypothesis is proposed:

**H1:**  *PSM positively influences the proactive service behaviors of grid workers.*

### 2.2. Occupational Identity and Proactive Service Behavior

In research on proactive behaviors, by studying the proactive behaviors of 622 managers in Australia, Parker and Collins classified proactive behaviors into three categories: proactive work behaviors, proactive strategic behaviors, and proactive person–environment (P–E) fit behaviors [30]. Proactive P–E fit behaviors refers to an individual’s initiative as to matching his/her attributes to the work environment and seeking change. This is based on the premise that the individual has conscientiousness about their job; in other words, the premise is that the individual identifies with the job. Tamir found that the greater an individual’s career identity, the more likely she or he is to actively engage in career management and P–E fit behaviors [31]. Therefore, we speculate that there may be a correlation between the occupational identity of grid workers and their proactive service behaviors.

Occupational identity or occupational identification refers to the extent to which individuals internalize their occupation as a valid definition of self; this is not limited to simple objective social categorization but is more oriented toward subjective identity perceptions from the heart [32]. This refers to autonomous perceptions of the individual based on a confluence of occupational attributes, values, beliefs [33], etc. Fugate and Kinicki found that individuals with high professional identity are better able to cope with changes in their work [34], which indirectly leads to higher personal performance and greater competitiveness [35,36]. At the same time, occupational identity will lead to an intrinsically positive experience of work, which has the effect of being an intrinsic motivator for individuals, meaning that they accept and enjoy their occupations to a greater extent [35]; this is likely to produce positive performance and proactive behaviors.

In the work context of Chinese grid workers, their duties require that they have a good sense of social service and the ability to work for the harmony and stability of the grid. When individuals choose this occupation, if their level of PSM is high, they are likely to be more inclined to believe in the value and significance of this occupation and truly categorize themselves as members of the occupational group. Therefore, the effect of their feelings or willingness to serve on their initiative will likely be transformed into a psychological state of active service, forming a solid sense of occupational identity. Once grid workers identify with the occupation, they may then adopt behavioral expressions that align with the occupational group, leading to positive active service. Therefore, we hypothesize that grid workers’ PSM can enhance their occupational identity and subsequently promote the emergence of proactive service behaviors:

**H2:**  *Grid workers’ occupational identity mediates the relationship between their PSM and proactive service behavior. PSM enhances the occupational identity of grid workers, which in turn enhances their proactive service behaviors.*

### 2.3. Impact of the Organizational Context

As noted, grid workers are present at the grassroots level; their work environment is complex, and they are the bridge between the government and the public. Therefore, in reality, they are susceptible to external organizational contextual factors. Whether in the private or public sector, employees’ organizational context will be an important influence on their behaviors. Crant noted the influences of organizational culture, organizational norms, situational cues, management support, public relations, and public or private settings [14]. We thus analyzed the situational factors that grid workers may face in their specific work context.

Grid workers in China are in a relatively uncertain situation due to the complexity of the grassroots environment. In normal times, the work of grid workers includes conflict investigation; however, in special times, their work may be temporarily increased to include more tasks, and this may become a complementary force or an additional aid to the government’s governance [3]. It is in this uncertain work environment that, most of the time, the job and role descriptions of grid workers are relatively unclear and lack task guidance, although these systems and standard processes are also in the process of being gradually explored and improved. Griffin found that an important psychological mechanism for proactive behavior is the elimination of uncertainty, and that employees are more likely to implement various types of proactive behaviors in uncertain environments [15]. Thus, they are better able to anticipate, understand, and influence their environment in advance, which in turn removes uncertainty and improves individual self-efficacy, forming a virtuous circle. Due to the uncertain environments in which grid workers find themselves, the discussion of organizational-level variables herein will focus on variables related to managerial support, as well as those related to organizational culture that fit the characteristics of the service area.

At the level of managerial support, scholars have found that leaders’ accountability to employees affects their willingness to engage in proactive behaviors and that, when leaders are accountable to their employees, employees tend to implement proactive behaviors to get their leaders’ attention [37]. This also suggests that seeking organizational support or organizational incentives will likely have an impact on employees’ proactive service behaviors. We sought to introduce the variable of organizational support to further portray the influence of managerial support factors on the proactive service behaviors of grid workers. In established research, scholars examining the sense of organizational support have found that it can be used to measure the extent to which individuals feel that their organization values their contributions and cares about their overall well-being [38]. In addition, we hypothesize that the organizational climate in which the grid workers are embedded may have an influential role on their proactive behavior.

We seek to portray this organizational climate through the concept of service climate. Schneider [39] defined service climate as a “description of what happens in people’s work units with regard to the service-focused policies, practices, and procedures they experience as well as the behaviors they observe being rewarded, supported, and expected.” In other words, it reflects the extent to which an organization values high-quality service. The service climate of an organization is the overall perception of the many measures that organization has in place to support high-quality service, as well as the behaviors they observe being rewarded, supported, and expected.

Both the sense of organizational support and the perceived service climate can have a positive impact on an individual’s positive affective commitment [40]. Employees will construct their occupational identity through interaction with the external environment on a personal and occupational basis, and their occupational identity will be influenced by the social environment, cultural climate, and management system [41]. Resource conservation theory suggests that when individuals have sufficient resources, such as goal-oriented motivational resources, experiential cognitive resources, and supervisor-supportive social resources [42], they will be inclined to invest in additional resources, make positive psychological identity commitments, and then produce positive behaviors. Good organizational incentives and a positive organizational service climate are the resources that grid workers can perceive, and these can positively influence the transformation of their motivation into psychological identity. From the perspective of organizational incentives, the spiritual motivation of superiors and the affinity and support of leaders can provide positive psychological resources to grid workers, while regular organizational training and publicity can help to improve the skills and experiences of grid workers.

In summary, organizational incentives and a positive organizational service climate can provide a positive organizational context for grid workers’ actual work and play a positive moderating role between their motivation and their occupational identity. Accordingly, the following hypotheses are proposed:

**H3:**  *Organizational support positively moderates the relationship between grid workers’ PSM and their occupational identity. Specifically, the higher the intensity of organizational support perceived by grid workers, the stronger the positive relationship between their PSM and occupational identity, and vice versa.*

**H4:**  *Organizational service climate positively moderates the relationship between grid workers’ PSM and occupational identity. Specifically, the stronger the organizational service climate perceived by grid workers, the stronger the positive relationship between their PSM and occupational identity, and vice versa.*

Hypotheses 1 and 2 illustrate the main effect of grid workers’ motivation on their proactive service behavior and the mediating role of occupational identity in this relationship, while hypotheses 3 and 4 illustrate the moderating role of the two types of organizational contexts in the relationship between personal motivation and occupational identity. The present study follows this logic and sees the need for a further moderated-mediation hypothesis, whereby the mediating role of grid workers’ occupational identities is moderated by the organizational context:

**H5:**  *Organizational support and organizational service climate at the organizational level moderate the mediating role of grid workers’ occupational identity between PSM and proactive service behavior. Specifically, the higher the level of organizational support and organizational service climate perceived by grid workers, the stronger the mediating role of occupational identity commitment.*

**Figure 1:**
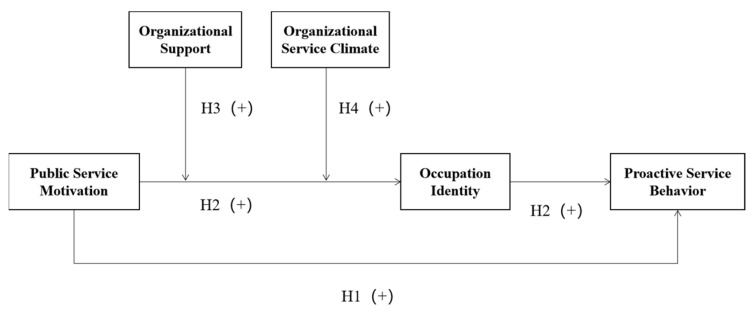
Variable relationship diagram (model diagram).

In summary, this study combines the findings of existing research to construct a mediation model of the effect of grid-worker PSM on proactive service behavior, in which occupational identity plays a mediating role, and organizational incentive support and organizational service climate are two moderating variables for the validity or otherwise of this mediation mechanism. Figure 1 shows the theoretical model of this study.

## 3. Materials and Methods

### 3.1. Measurements

The measurement tools used in this study are the more mature scales that have been applied to this topic worldwide, and the relevant questions were modified based on specific scenarios. All latent variables were scored on a seven-point Likert scale, with 1 indicating “strongly disagree”, 2 indicating “disagree”, 3 indicating “somewhat disagree”, 4 indicating “neutral”, 5 indicating “somewhat agree”, 6 indicating “agree”, and 7 indicating “strongly agree”. Before the variables were measured, to ensure that the scales were valid in the Chinese context, we conducted in-depth interviews with a random sample of 36 grassroots grid workers in the eastern region, and some of the questions relating to the variables were slightly revised in the Chinese context.

#### 3.1.1. PSM

The Public Service Motivation Scale was developed by Kim in 2013 [43]. It contains a total of four dimensions corresponding to 16 question items, including attraction to public policymaking, commitment to civic duty and the public interest, compassion, and self-sacrifice. Through interviews, we found that, in the Chinese context, due to the job requirements and responsibilities set for Chinese grid workers in social governance, they are the group closest to the grassroots people; as such, serving and assisting are their main responsibilities, and these do not involve the policymaking aspect. Therefore, the PSMs of grid workers in this survey mainly reflect commitment to civic duty and the public interest, compassion, and self-sacrifice, but they do not include attraction to public policymaking.

#### 3.1.2. Occupational Identity

Occupational identity, as considered in this study, also has a well-established scale: the Occupational Identity Scale developed by Mael and Tetrick [44], which has six items.

#### 3.1.3. Organizational Support

To measure organizational motivation, this study used a conceptually close maturation scale related to perceptions of organizational support: the Organizational Support Scale developed by Hayton in 2012 [45], which consists of four question items. These question items were formulated more concretely according to the context in which the grid workers are located, making it easier for the people completing it to understand the distinctions. For example, the item, “The organization values my contribution to its well-being” in the original scale changes to “The organization values my contribution to its well-being, and my leader will give me spiritual horns and commendations”.

#### 3.1.4. Organizational Service Climate

Organizational service climate also has a well-established scale: the Perceived Service Climate Scale, developed by Schneider in 1998, which consists of seven items [39,46]. The original questions, such as, “How would you rate the company’s efforts to measure and track the quality of its services?” and, “How would you rate the recognition and rewards received by its employees for delivering quality services?” with “companies” replaced by “grids” and “customers” replaced by “people” can also be effectively matched to the content of the questions prepared for this study. Based on the pre-interviews with the grid workers, the formulation of the items was changed from questions to statements, which made it easier for the fillers to reply.

#### 3.1.5. Proactive Service Behavior

The Proactive Service Behavior Scale developed by [9] consists of seven items. Some of the questions of on this scale were adapted and optimized, making them more relevant to the Chinese context and the specific working environment of grid workers. For example, in the original scale, “My staff member proactively shares information with customers to meet their financial needs” was modified to, “I will actively respond to the needs of the grid residents and do my best to meet and serve them”. In the original scale, “My staff member uses their own judgment and understanding of risk to determine when to make exceptions or improvise solutions” was revised to, “I will take the initiative to do a good job of preventing problems beforehand and visit the grid in time to resolve potential hidden dangers and minor conflicts” in this study.

#### 3.1.6. Control Variables

Following the custom of previous studies, the control variables in the present study were gender, age, education, occupation type, work area, Communist Party of China (CPC) member status, social worker status, and working experience in the organization, all of which were filled in by the grid workers according to their situation. In the collected data, the variables are defined as follows. Gender is set as “1” for male and “0” for female. Age is the actual number for each grid worker. Education is divided into four categories, “1” to “4”, including high school and below, specialty, bachelor’s degree, and postgraduate and above. Occupation type is divided into two categories: full-time grid workers are recorded as “1”, and part-time grid workers are recorded as “0”. Work areas are also divided into two categories: urban grid workers are recorded as “1”, and a value of “0” is used for rural grid workers. In the case of CPC membership and part-time social worker status, “1” means yes and “0” means no. Years of work experience was collected by participants filling in the year in which they officially became a grid worker and subtracting it from the current year. In the following statistics, we will estimate the impact of these control variables through stratified regression.

### 3.2. Research Sample

To enhance the reliability and validity of the content of the scale, a pre-survey was conducted with a small sample before formally distributing the questionnaire to the large sample. Through the sending of a link, a total of 61 in-service grassroots grid members were invited to complete an online questionnaire; the reliability and validity of the questionnaire items were then tested accordingly. The basic information of the 61 grid workers who participated in this pre-survey are shown in the Table 1.

Due to the adaptation of the scale in a Chinese context, we will use methods such as exploratory factor analysis to test whether the adapted scale has good internal consistency. As shown in Table 2, the Kaiser–Meyer–Olkin (KMO) test value of each scale was greater than 0.7, and the *p* value of Bartlett’s test was 0.000. Therefore, it was deemed suitable for factor analysis. The results of exploratory factor analysis showed that the factor loading value of each scale question item was above 0.6, and Cronbach’s α was greater than 0.8, indicating that the initial questionnaire had good internal consistency, according to which the formal questionnaire was formed.

After the pre-survey questionnaire recovery and scale test was completed, the formal questionnaire survey was started. The sample object was the group of grid workers working at the grassroots front line. To efficiently obtain the sample of grid workers and improve the quality of the answers, local government departments and other channels were entrusted to carry out the distribution of the electronic questionnaire in February 2023. The questionnaire was in electronic form, and it could be accessed through an electronic link or QR code. The subjects accessed the questionnaire on their mobile devices and completed it alone. Before the formal completion of the questionnaire, each grid worker was given background information about the study and was assured that his/her personal information would not be disclosed. Finally, all participants filled in their names to facilitate the checking and matching of data processing at a later stage.

To achieve ex ante control of common method bias (CMB) and reduce the impact of common method variance (CMV) on the results of the study, the questionnaire distribution was divided into two rounds with a time interval of about one week. In the first round of the survey (T1), one independent variable (PSM) and two moderating variables (organizational incentives and positive organizational climate) with control variables were collected. One week after T1, the second survey (T2) collected the mediator variable (occupational identity) with the dependent variable (proactive service behaviors). Because all participants’ identities were unique and both rounds of questionnaire collection requested that subjects fill in their names, names could be used to match the data from both rounds for each grid worker. In terms of the specific recovery volume, 436 questionnaires were collected in the T1 stage and 428 questionnaires were collected in the T2 stage. After two rounds of data matching and excluding data that were invalid (filling out the questionnaire over too short a period or all the answers being the same), we finally obtained 348 valid samples that were successfully matched in two rounds of data matching. This represents an effective recovery rate of more than 75%. The characteristics of the sample in the formal surveys are shown in Table 3.

By measuring the two time periods of the sample, we will analyze the collected data. Firstly, we will conduct confirmatory factor analysis using Mplus 8.10 to discuss the reliability and validity of the data. On the basis of ensuring reliability and validity, we used SPSS 25 and the PROCESS program to sequentially test the direct effect, mediating effect, moderating effect, etc. in the model.

## 4. Results

### 4.1. CMV

Two approaches were used to reduce the interference of CMB on the analysis of quantitative results: “ex ante” and “ex post” test control. For the ex ante control, the questionnaire was distributed in two phases and matched, and all of the questions for a single variable were set on the same page. Furthermore, all the questions for each variable had to be answered before turning the page to fill in the questions of the next variable; this can reduce the severity of the CMB to a certain extent. Harman’s one-factor test was used to determine whether there was a serious CMB. Using the SPSS 25 package, it was found that the variance contribution of the first factor was 42.397%, which is less than 50% and thus below the 50% criterion for determination [47].

### 4.2. Reliability and Validity Tests

The internal consistency of the variables was examined through Cronbach’s α value, compositional reliability (CR), and average variance extraction (AVE) (see Table 4). As can be seen from the table, the Cronbach’s α values of all variables were above 0.8 and even reached above 0.9; furthermore, the CR values of all variables were above 0.9 and, similarly, most of the variables exceeded 0.95; lastly, the AVE values of all variables exceeded the acceptable criterion of 0.36, and all of them reached the desirable level of above 0.6. In conclusion, the internal consistency of the variables used in this study was high and it thus has good reliability. Then, we used Mplus 8.10 during the formal survey phase to conduct confirmatory factor analysis on the validity of each variable and the overall model. As shown in Table 5, the overall measurement model and the fitting results of each variable are relatively ideal. The fitting indicators of each scale meet the judgment criteria, indicating that the scale has good structural validity (χ^2^/df = 2.828, CFI = 0.911, TLI = 0.909, RMSEA = 0.081, SRMR = 0.073).

The correlation analysis results indicate that the independent variable of PSM is significantly positively correlated with the mediating variable of occupational identity, as well as with the dependent variable of proactive service behavior (see Table S1). In addition, there is a significant positive correlation between the moderating variables and the mediating and dependent variables. This provides a preliminary direction and basis for hypothesis testing.

### 4.3. Direct Effect and Mediating Effect

In this study, the direct and mediating effects were tested using two methods: stepwise regression and bootstrap analysis. First, the mediation-effect test procedure of the stepwise regression method, and the linear regression analyses of the control, independent, mediating, and dependent variables were conducted using SPSS 25; the results are shown in Table 6. In this case, the value of the variance inflation factor (VIF) of each model is less than 5, which indicates that the problem of multicollinearity of the variables under consideration in this study is not serious.

As shown in Table 6, Model 1 contains only the effect of the control variables on the mediating variable, occupational identity, to which Model 2 adds the independent variable, PSM. It can be found that PSM has a significant positive effect on occupational identity (β = 0.461, *p* < 0.001), and the model explanation is significantly greater (ΔR^2^ = 0.186, *p* < 0.001). Model 3 includes only the effect of the control variable on the dependent variable, proactive service behavior, and Model 4 adds the independent variable PSM to this. It can be seen that there is a significant positive effect of PSM on proactive service behavior (β = 0.157, *p* < 0.01), and the degree of model explanation is significantly improved in Model 4 compared to Model 3 (ΔR^2^ = 0.027, *p* < 0.01). Hypothesis 1 is thus accepted. This means the PSM of grid workers can stimulate their proactive service behaviors.

For Model 5, the mediator variable, occupational identity, was added based on Model 4, in which occupational identity had a significant positive effect on proactive service behaviors (β = 0.313, *p* < 0.001), and PSM had a positive but not significant effect on proactive service behaviors. The degree of explanation of Model 5 was again significantly higher than that of Model 4 (ΔR^2^ = 0.082, *p* < 0.001). Considering the first two steps in combination, it can be seen that occupational identity plays a fully mediating role between PSM and proactive service behavior, and Hypothesis 2 is thus accepted. As the results show, the PSM of grid workers is enhanced by improving their occupational identity, which in turn stimulates their proactive service behavior.

This study also used the PROCESS program to conduct a bootstrap (set to 5000) analysis of the mediating effect of occupational identity on the independent variable of PSM and proactive service behaviors, with a confidence interval of 95%. All the research variables were centered before analysis, and the results are displayed in Table 7. In terms of the main effect, there is a positive relationship between PSM and proactive service behaviors, with the confidence interval not containing 0 and reaching a significant level. This again confirms the validity of Hypothesis 1. Regarding the mediating effect, the coefficient of the indirect effect of the mediating variable occupational identity, in the case of the independent variable PSM, is significantly positive. This indicates that occupational identity plays a mediating role between PSM and proactive service behaviors, and Hypothesis 2 is again shown to be valid. The Sobel test was used to show that the mediating effect of occupational identity does exist, and it was found that the coefficients are all positive. Furthermore, the Z value is 4.924, which is greater than 2.58, and this reaches a significance level of 99%. This is further validation of the mediating effect of occupational identity, and the PSM of grid workers can thus be said to enhance their level of occupational identity, which in turn generates more proactive service behaviors.

### 4.4. Moderating Effect

This study continued with a bootstrap (set at 5000) analysis of the moderating effects of organizational support and organizational service climate between the independent variable of PSM and the mediating variable of occupational identity using the PROCESS program with a 95% confidence interval, and the results are shown in Table 8.

**Figure 2:**
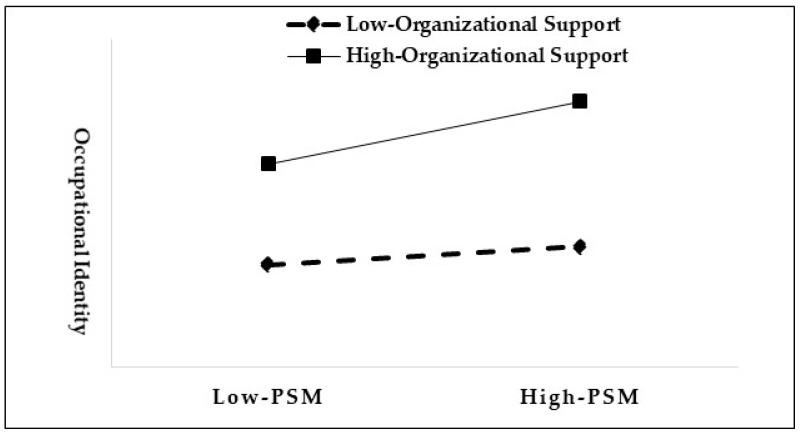
Moderating effect of organizational support on PSM and occupational identity.

Regarding the moderating effect of organizational support, the statistical results show that the coefficient of the interaction term between PSM and organizational incentives is 0.121; the 95% confidence interval does not contain 0 and reaches a significant level, which indicates that there is a moderating effect of organizational incentives on PSM and occupational identity. As shown in Figure 2, when the level of organizational incentives perceived by the grid workers is high (M + SD), the coefficient between PSM and occupational identity is 0.332, and the confidence interval does not contain 0 and reaches the required significance level; in the case of a low level of organizational incentives perceived by the grid workers (M − SD), the coefficient drops to 0.099 and is not significant. This result suggests that the higher the level of perceived organizational support of the grid workers, the more their PSM enhances their commitment occupational identity, thus Hypothesis 3 is proved.

**Figure 3:**
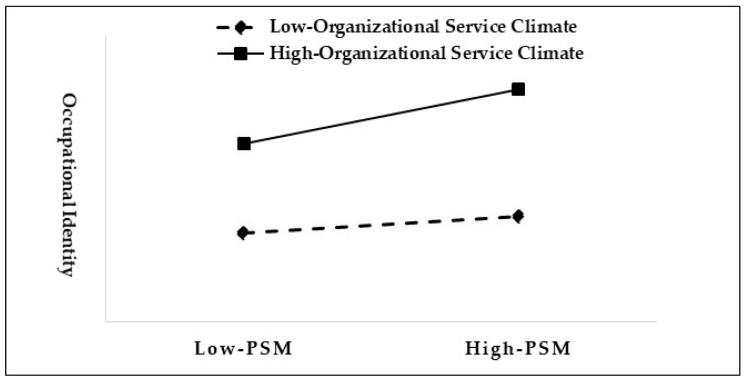
Moderating effect of organizational service climate on PSM and occupational identity.

Regarding the moderating effect of organizational service climate, the coefficient of the interaction term between PSM and organizational service climate is 0.065, which does not contain 0 at the 90% confidence interval and reaches the required significance level. This indicates the existence of a moderating effect of organizational service climate on PSM and occupational identity. The moderating effect is shown in Figure 3, in which the coefficient between PSM and occupational identity is 0.219 when the level of organizational service climate perceived by grid workers is high (M + SD), and the confidence interval does not contain 0, reaching the required significance level; the coefficient decreases to 0.089 when the level of organizational service climate perceived by grid workers is low (M − SD), which is also significant. These statistical results indicate that the higher the level of the perceived organizational service climate of the grid workers, the more their PSM enhances their occupational identity; Hypothesis 4 is thus proved.

### 4.5. Moderated Mediation Effects

The above hypothesis tests were conducted to test the mediating effects of occupational identity between personal motivation variables and proactive service behaviors, and the moderating effect of organizational incentives and organizational service climate in a localized way. All hypotheses were able to be established or supported by the data. Next, the complete path from PSM to proactive service behavior was tested; index values and subgroup analysis results are shown in Table 9.

PSM influences the path of action of occupational identity commitment, which in turn influences proactive service behavior. When organizational motivation was used as a moderating variable, the index value was 0.038 with a 95% confidence interval of [boot LLCI = 0.013, boot ULCI = 0.078], which does not contain 0, indicating that the mediating effect of the moderated effect does exist. Specifically: when the grid workers’ perceived level of organizational incentives is low, the mediating effect of commitment to occupational identity is only 0.031 and the 95% confidence interval contains 0, and this is not significant; when the grid workers’ perceived level of organizational incentives is medium, the mediating effect of occupational identity is 0.067, which is not only an elevated coefficient but also reaches the level of significance as the 95% confidence interval does not contain 0; and when the grid workers’ perceived level of organizational motivation is high, the mediation effect of occupational identity is 0.104, which is the highest among the three groups, and the 95% confidence interval does not contain 0, which is significant. When organizational service climate is used as a moderating variable, the index value is 0.020 with a 95% confidence interval of [boot LLCI = 0.001, boot ULCI = 0.048], which does not contain 0, indicating that the moderated mediation effect does exist.

Furthermore: when the level of organizational service climate perceived by the grid workers is low, the mediating effect of occupational identity is only 0.028 and the 95% confidence interval contains 0, which is not significant; when the level of organizational service climate perceived by the grid workers is medium, the mediating effect of occupational identity is also elevated to 0.067 and the 95% confidence interval does not contain 0, which reaches the level of significance; and when the level of organizational service climate perceived by grid workers is high, the mediating effect of occupational identity is 0.104, which is the highest among the three groups and the 95% confidence interval does not contain 0, and this result is significant.

Considering the above index-value determination and analysis results in combination, it is not difficult to find that the moderated mediation effect does exist, and the mediation effect of the grid workers’ occupational identity between her/his PSM and proactive service behaviors will be moderated by the organizational context variables: the higher the level of organizational incentive support perceived by the grid worker, the stronger the mediation effect of the commitment to occupational identity between an individual’s PSM and their proactive service behavior. The higher the level of perceived organizational service climate, the stronger the mediating effect of occupational identity between an individual’s PSM and their proactive service behavior. For the mechanism of the influence of PSM, the mediating effect is supported by both index values and subgroup analyses, and Hypothesis 5 is thus proved.

## 5. Discussion

In this study, the proactive service behaviors of a group of grid workers in eastern China was studied, and the mechanisms influencing the proactive service behaviors of these grid workers were explored. It was found that the PSM of grid members is a positive and important individual motivation that can effectively stimulate their proactive service behavior. Based on these results, it can be said that grid workers mainly show proactive behaviors individually based on their PSM. Second, the occupational identity of grid workers plays a positive mediating role in the above path. Individual grid workers with PSM can continuously increase their identification with their duties in their actual work, which in turn contributes to the emergence of proactive service behaviors. Meanwhile, organizational incentives and organizational service atmosphere are important moderators of the effective transformation of motivation into psychological identity. The transformation of an individual’s motivation to commitment to occupational identity is an intra-organizational process, during which the necessary organizational support and service atmosphere become important factors to deepen this transformation. Finally, in terms of the complete mediating effect, the mechanism by which a grid worker’s PSM enhances active service behavior by improving occupational identity is positively moderated by organizational incentives and organizational service climate. In other words, the higher the levels of these two types of positive organizational context, the stronger the mediating effect of grid workers’ occupational identity will be.

This study tested the mediating role of occupational identity between grid workers’ PSM and individual proactive service behaviors. The results explain that an important prerequisite for proactive service behaviors among grid workers in China is identification with their profession, in other words, dedication to their work. Similar to the findings of studies related to occupational identity, groups with high occupational identity can be more actively engaged in their occupational development [48], and this more positive attitude is an important antecedent variable for proactive service behaviors among the grid-worker group.

We verified the important mediating role of occupational identity in the pathway between individual motivation and proactive behavior, as well as the important moderating role played by organizational contextual variables including organizational incentives and organizational service climate. These findings extend the proactive behavior model constructed by Crant (2000). As the antecedent variables of proactive behavior in the model constructed by Crant contain both individual characteristics and organizational context, our study verifies the important role of organizational contextual factors. It is worth noting that this work reveals the chain of action between the two types of factors in proactive behavior. Individual factors need to be further motivated by occupational identity to stimulate proactive behavior, while organizational context plays more of a moderating effect. In the original model of proactive behavior, Crant (2000) suggested that organizational factors may lead to the generation of context-specific behaviors such as seeking feedback, proactive socialization, and innovative behaviors. Although relevant variables were not constructed for testing in the present study, we are more inclined to believe that the generation of proactive behaviors among Chinese grid workers is not only affected by the motivation for public service and professional identity factors. We regard such proactive behaviors as a kind of specific behavior generated from a sufficient mixture of situational and personal factors.

There are still some questions that are worth continuing to explore, such as whether there are still other mediating variables in the proactive service behaviors of groups such as grid workers, especially psychological factors, in addition to the elements of occupational identity, or positive affective elements. In other works, organizational identity has also been found to be an important variable influencing individuals’ behavior, especially as the concept of organizational identity has been associated with concepts such as occupational identity and high performance [49,50]. In addition, related variables such as personal political orientation [51], occupational reputation [32], and organizational reputation [52], all have the potential to play a role in the relationship between personal motivation and organizational identification, as well as in the relationship between organizational identification and proactive behaviors, all of which needs to be further explored.

In terms of contextual factors, limited by the research design and sample characteristics, this study did not examine the moderating effects of different types of contexts, such as the public and private sector, on whether there will be a moderating effect between an individual’s occupational identity and his or her proactive service behaviors. Indeed, testing this set of relationships could help to further refine the proactive behavior model, clarify the specific associations between proactive service behaviors and organizational situational factors, and identify the possible organizational conditions under which proactive service behaviors are generated among this group of grid workers.

### 5.1. Theoretical Significance and Practical Implications

Research examining the proactive service behaviors of grid workers in China can help to further enhance the discussion and accumulation of knowledge on the factors influencing proactive service behaviors, especially by expanding the research from formal employees in the private and public sectors to different groups such as informal employees in the public sector. This could help to expand the scope of the application of proactive service behaviors as a theory. In established research, scholars have called for research on proactive behaviors in new contexts, as well as for a process theory of proactive behaviors [14]. This study adds a new context of grassroots grid governance in China. Although this context is also not outside the realm of individual-organizational factors in the proactive behavior model, in this context, we found the role of organizational incentives and organizational service climate in moderating the relationship from individual motivation to occupational identity. This revealed the mechanisms by which proactive service behaviors are generated in Chinese grid members at the micro level.

From the perspective of the target group, this study focused on grid workers, who can provide services on their initiative despite their multiple job responsibilities. This is of great significance to grass-roots social governance in China. Research on grid workers is dominated by expository articles, and little empirical work has been conducted on them as research objects; theoretical research and real-life practice have not paid sufficient attention to the specific work and lives of grid workers. It should be noted that some scholars hold a negative view of grid workers and grid governance, arguing that they are more of a top-down monitoring and controlling tool than a service provider, and that they may even weaken the participation of the general public in governance [53]. However, the findings of this study are consistent with the fact that—both in the toughest moments of the epidemic and in their daily work—grid workers have effectively fulfilled their service-orientated role and have become the bottom-up guarantee of epidemic prevention and relief and stable development within the grids. Through empirical analyses, it is hoped that this study demonstrates that the proactive service of grid workers stems from their public service motives, thus highlighting the nature of Chinese grid governance as a service to the grassroots, providing a counterpoint to the assertion that the grid is “supervising and controlling, hindering the governance of the people” (监管控制, 阻碍民治). These findings thus indicate the need for more theoretical and practical attention to be paid to the group of grid workers, giving them understanding and support.

The relevant findings of this study will help scholars better understand the work status of Chinese grid workers. As mentioned in the introduction, we cannot ignore the important role of grid workers in grassroots governance in China, especially in terms of stability. At the same time, we cannot ignore their sustainable development just because they are a new professional group. In this study, we indirectly revealed the work status of grid workers from the perspective of proactive service behavior. A very important point is that, although grid workers have a lot of work tasks, their public service spirit and recognition of their profession keeps them enthusiastic about their work and showing proactive service behavior. At the same time, the organization where the grid members are located, the grid management organization, has a motivating effect on the grid members while maintaining a good service atmosphere. Therefore, in this study, we indirectly discovered the relationship between the work status of Chinese grid workers and personal and organizational factors. Based on the findings of this study, we are more inclined to believe that, in order to further promote the sustainable development of the professional group of grid workers in China, in addition to screening personnel with strong public service motivation, more organizational incentives are still needed at the organizational level to shape a better organizational atmosphere and promote grid workers to achieve stronger professional identity, thereby bringing good work results and peripheral effects, that is, proactive service behavior. Based on the proactive service behavior of Chinese grid workers during the COVID-19 pandemic, we also hope to promote the sustainable development of this occupational group through a series of studies, which will help China achieve the United Nations Development Goal of reducing global health risks (see SDG-3D).

Our research findings provide a space for discussion in promoting the development of emerging occupational groups and sustainable work research. In their research on social sustainability in the workplace, Grum and Babnik reviewed relevant literature and found that Sustainable job/work characteristics include three themes: decent work, meaningful work, and sustainable work [54]. Based on our research, we call for more exploration to maintain the proactive service behaviors generated by grid workers in China and promote the stability and sustainable development of this good professional group in China, including but not limited to discussions on decent work, job happiness, and other aspects. Returning to our research background, our research also echoes the sustainable development goals of the United Nations, particularly the aim to “achieve full and productive employment and deep work for all women and men, including young people and individuals with disabilities, and equal pay for work of equal value” (SDG-8.3). We also call on scholars to conduct OB-related research on various emerging occupational groups, and pay attention to and explore the behavior and sustainability of their work, in order to better help achieve the United Nations Sustainable Development Goals.

### 5.2. Research Limitations and Future Prospects

This study has some limitations, which will now be discussed. First, both at the interview stage and the questionnaire-distribution stage, the selection of the sample was limited to a certain extent. This is manifested in the sampling method, sample characteristics, and geographical location. Because of the limited number of grassroots grids that could be contacted, it was difficult to achieve strict random sampling. As such, in the final sample, there were fewer male grid workers and fewer rural grid workers, and all of them came from Province Z in eastern China, making the sample less representative. The validity of the findings in other provinces and cities is thus unknown, and the external validity of the findings is consequently affected. Because of the limited research resources and the need to match the questionnaires between the two phases, the final sample size of 348 that could be analyzed was relatively limited. In the future, if sufficient research resources and channels are available, the sample could be further expanded into multiple regions, the sampling process could be optimized, and the adaptability of the findings in different regions could be tested.

Second, although the questionnaire was distributed in two time periods and the method of measuring different variables on separate pages was used to reduce the effect of CMV, it is unfortunate that it was not completed by a wider range of different evaluation subjects. We followed the path of “motivation tendency behavior” and indirectly measured the proactive service behavior of grid workers, but this measurement method actually has limitations. We hope that, in future research, scholars can develop workbooks or more focused and standardized scales to observe proactive service behavior, in order to advance related research in this field. In addition, as the measurement questions were mostly positive, subjects may have tended to choose scores in self-assessment under the influence of social desirability, resulting in distortion of data and bringing bias into subsequent analyses. Future research should be based on the availability of appropriate resources and channels, and the questionnaires should be filled out by multiple subjects at multiple points in time to improve the validity and authenticity of the questionnaires and thus enhance the reliability of the data analyses.

Therefore, in future research, differences in the antecedent mechanisms of proactive service behaviors can be examined for different types and traits of grid-worker groups. Among grid workers, there are not only gender, type, and urban–rural differences, but also differences in the level of regional development. Within this, it is worth exploring how the level of proactive service varies across different regions of China and whether the role played by their motivations is consistent with the findings of this study. In the future, a sample of grid workers could be selected from East, Central, and West China, their proactive service behaviors and corresponding levels of antecedent variables could be measured. This would not only test the external validity of the results of the present study but also explore in depth the mechanism of variation conferred by regional differences. This would provide a better understanding of the connotations of the proactive service behaviors of grid workers and the factors influencing them.

Third, empirical research on the “weak proactive service behavior” of grid workers could be conducted. In recent years, reflections on proactive service behaviors have not ceased; in the most representative of these, it was found that proactive service provision may not only create surprises for service recipients but could also bring about service redundancy [55]. This is not only unnecessary but also reduces the customer’s perception of service [56,57], and it could even bring about increases in organizational costs and resource wastage [57]. Therefore, some scholars have proposed the concept of weak proactive service behavior, which is defined as “an employee’s ability to stand in the customer’s shoes and provide appropriate and delicate proactive service to the customer after accurately understanding the customer’s potential needs laterally during the service process,” with the characteristics of “spontaneity, accuracy, and empathy” [58]. In the grid-worker group, it is also possible to dig deeper into these weak proactive service behaviors through interviews with both grid workers and grid residents. They could be asked for their perceptions of and views regarding proactive service behaviors, and the differences in perceptions between the two could be compared to determine the boundaries of proactive service behaviors in terms of accuracy, necessity, and inappropriateness. The results of this work will help grid workers to identify unnecessary service behaviors and improve proactive services more appropriately, reducing the burden while increasing the satisfaction level of service recipients, thus achieving the beneficial effect of getting twice the result with half the effort.

## Figures and Tables

**Table 1:** Demographic characteristics of the initial questionnaire.

Category	Classification	Frequency (%)	Category	Classification	Frequency (%)
Gender	Male	38 (62.3%)	Working area	Urban areas	39 (63.9%)
	Female	23 (37.7%)		Rural areas	22 (36.1%)
Age	Under 25	4 (6.6%)	Member of the CPC	Yes	20 (32.8%)
	26–35	31 (50.8%)		No	40 (67.2%)
	36–45	26 (42.6%)	Social worker status	Yes	9 (14.8%)
	Beyond 46	0		No	52 (85.2%)
Education	High school and below	10 (10.6%)	Work experience	2 years and less	5 (8.2%)
	Specialty	27 (44.3%)		3 to 5 years	43 (70.5%)
	Bachelor’s degree	23 (37.7%)		6 to 10 years	13 (21.3%)
	Postgraduate and above	1 (1.6%)		10 years and above	0
Occupation type	Full-time	48 (78.7%)			
	Part-time	13 (21.3%)			

**Table 2:** Results of exploratory factor analysis of the initial questionnaire.

	PSM	Organizational Support	Organizational Service Climate	Occupational Identity	Proactive Service Behavior
KMO	0.939	0.886	0.774	0.823	0.962
Bartlett’s test	0.000	0.000	0.000	0.000	0.000
Cumulative % of variance	70.121%	78.595%	76.345%	65.334%	78.358%
Minimum factor loading	0.616	0.740	0.692	0.707	0.683
Cronbach’s α	0.943	0.910	0.815	0.874	0.969

**Table 3:** Demographic characteristics of formal survey sample.

Category	Classification	Frequency (%)	Category	Classification	Frequency (%)
Gender	Male	128 (36.8%)	Working area	Urban areas	245 (70.4%)
	Female	220 (63.2%)		Rural areas	103 (29.6%)
Age	Under 25	21 (6.0%)	Member of the CPC	Yes	185 (53.2%)
	26–35	151 (43.4%)		No	163 (46.8%)
	36–45	128 (36.8%)	Social worker status	Yes	151 (43.4%)
	Beyond 46	48 (13.8%)		No	197 (56.6%)
Education	High school and below	32 (9.2%)	Work experience	2 years and less	106 (30.4%)
	Specialty	99 (28.4%)		3 to 5 years	127 (36.5%)
	Bachelor’s degree	210 (60.3%)		6 to 10 years	67 (19.3%)
	Postgraduate and above	7 (9.2%)		10 years and above	48 (13.8%)
Occupation type	Full-time	279 (80.2%)			
	Part-time	69 (19.8%)			

**Table 4:** Results of the reliability analysis of the formal survey scale.

Variable	M	SD	Cronbach’s α	CR	AVE	KMO	Bartlett’s Test
PSM	5.71	1.15	0.938	0.937	0.605	0.924	0.000
Organizational support	5.59	0.96	0.885	0.945	0.657	0.870	0.000
Organizational service climate	5.61	1.00	0.836	0.952	0.768	0.789	0.000
Occupational identity	5.59	1.15	0.934	0.936	0.677	0.901	0.000
Proactive service behavior	5.62	1.04	0.950	0.958	0.698	0.945	0.000

**Table 5:** Overall model and coefficient-of-fit results for each scale.

Variable	χ^2^/df	CFI	TLI	RMSEA	SRMR
Overall	2.828	0.911	0.909	0.081	0.073
PSM	3.684	0.915	0.904	0.083	0.076
Organizational support	4.226	0.945	0.907	0.076	0.051
Organizational service climate	3.544	0.951	0.918	0.091	0.077
Occupational identity	3.152	0.949	0.904	0.084	0.052
Proactive service behavior	3.504	0.935	0.910	0.080	0.046

**Table 6:** PSM and proactive service behavior: the mediating effect of occupational identity.

Variables	Occupational Identity		Proactive Service Behavior		
	Model 1	Model 2	Model 3	Model 4	Model 5
Constant	3.796 ***	1.940 ***	4.460 ***	3.827 ***	3.220 ***
Gender	−0.555 ***	−0.337 **	−0.161	−0.087	0.018
Age	0.036 ***	0.020 *	0.022 *	0.016	0.010
Education	0.120	0.065	0.029	0.010	−0.010
Occupation type	0.169	0.181	0.012	0.016	−0.040
Work area	0.371 *	0.288 *	0.258	0.230	0.140
CPC membership	0.105	0.041	0.045	0.023	0.010
Social worker status	−0.025	−0.008	0.245 *	0.250 *	0.253 *
Work experience	−0.017	−0.020	0.011	0.002	0.008
PSM		0.461 ***		0.157 **	0.013
Occupational Identity					0.313 ***
R^2^	0.132	0.318	0.064	0.091	0.148
ΔR^2^	0.132 ***	0.186 ***	0.064 **	0.027 **	0.082 ***
F	6.416 ***	17.482 ***	2.909 **	3.750 ***	7.025 ***

Notes: The sample size is 348. * *p* < 0.05, ** *p* < 0.01, *** *p* < 0.001.

**Table 7:** Bootstrap analysis of main effects and mediating effects.

Independent Variable	Effect	Coefficients	Boot LLCI	Boot ULCI
PSM	Main effect	0.157	0.059	0.255
	Direct effect	0.013	−0.093	0.119
	Indirect effect	0.144	0.064	0.232
	R^2^	F	Sobel test	
	0.173	7.025 ***	0.144 ***	Z = 4.924

Notes: The sample size is 348. *** *p* < 0.001.

**Table 8:** Results of moderating-effects analysis.

Approach	Results			
PSM ↓ Occupational identity	Moderating variable: Organizational support		Interaction term coefficients: 0.121 **	
	Moderation level	Coefficient	LLCI	ULCI
	Low	0.099	−0.001	0.199
	Middle	0.215	0.134	0.296
	High	0.332	0.213	0.450
	Moderating variable: Organizational service climate		Interaction term coefficients: 0.065 +	
	Moderation level	Coefficient	LLCI	ULCI
	Low	0.089	0.002	0.176
	Middle	0.154	0.077	0.231
	High	0.219	0.100	0.338

Notes: Sample size is 348; + *p* < 0.1, ** *p* < 0.01; In the moderated level section, Low = M − SD, Middle = M, High = M + SD.

**Table 9:** Moderated mediation effects.

Approach	Results			
PSM ↓ Occupational identity ↓ Proactive service behavior	Moderating variable: Organizational support		Index: 0.038 **	
	Moderation level	Mediation effect coefficient	Boot LLCI	Boot ULCI
	Low	0.031	−0.0038	0.079
	Middle	0.067	0.0211	0.112
	High	0.104	0.0304	0.167
	Moderating variable: Organizational service climate		Index: 0.020 *	
	Moderation level	Mediation effect coefficient	Boot LLCI	Boot ULCI
	Low	0.028	−0.0045	0.075
	Middle	0.048	0.020	0.085
	High	0.068	0.031	0.113

Notes: Sample size is 348. * *p* < 0.05, ** *p* < 0.01. In the moderated level section, Low = M − SD, Middle = M, High = M + SD.

## Data Availability

The data that support the findings of this study are available on request from the corresponding author. The data are not publicly available due to privacy or ethical restrictions.

## Supplementary Materials

The following supporting information can be downloaded at: https://www.mdpi.com/article/10.3390/bs14030148/s1.
