# Supplementary material for: How Does Public Service Motivation Affect the Proactive Service Behaviors of Grid Workers? A Study of Survey Evidence from Eastern China
Source: Behav Sci (Basel). 2024 Feb 20;14(3):148. doi: 10.3390/bs14030148 (PMC10967981; doi:10.3390/bs14030148)
Supplement: Supplementary file 1 [file behavsci-14-00148-s001.zip › behavsci-2786968-supplementary.pdf]

Supplementary Materials

Table S1. Correlation coefficients between the main variables.

Variable	1.	2.	3.	4.	5.
1. PSM	(0.778)				
2. Organizational Support	0.491**	(0.811)			
3. Organizational service climate	0.508**	0.577**	(0.876)		
4. Occupational Identity	0.521**	0.537**	0.604**	(0.823)	
5. Proactive service behavior	0.222**	0.703**	0.604**	0.380**	(0.835)

Notes: Sample size is 348. ** p <0.01. The square roots of the variable AVE are presented in brackets in the diagonal cells.
